# Antihyperglycemic effect of rice husk derived xylooligosaccharides in high‐fat diet and low‐dose streptozotocin‐induced type 2 diabetic rat model

**DOI:** 10.1002/fsn3.1327

**Published:** 2019-12-09

**Authors:** Nuntawat Khat‐udomkiri, Parichart Toejing, Sasithorn Sirilun, Chaiyavat Chaiyasut, Narissara Lailerd

**Affiliations:** ^1^ Innovation Center for Holistic Health, Nutraceuticals and Cosmeceuticals Faculty of Pharmacy Chiang Mai University Chiang Mai Thailand; ^2^ Department of Physiology Faculty of Medicine Chiang Mai University Chiang Mai Thailand

**Keywords:** antihyperglycemia, diabetes mellitus, gut microbiota, prebiotic, rice husk, xylooligosaccharides

## Abstract

Rice husk (RH) is an agricultural waste obtained from rice milling process. Our previous study demonstrated the optimized process of extracting xylooligosaccharides (XOS), a prebiotic that can support the growth and activity of beneficial gut microbiota, from RH. Accumulated evidences indicate that the composition of gut microbiota is involved in the progression of insulin resistance and diabetes. This study aims to evaluate the antihyperglycemic effect and putative mechanisms of RH‐XOS using a diabetic rat model induced by high‐fat diet and streptozotocin injection. Diabetic rats were randomly assigned to receive vehicle (DMC), XOS (DM‐XOS), metformin (DMM), and a combination of XOS and metformin (DMM‐XOS). An additional group of rats were fed with normal diet plus vehicle (NDC) and normal diet plus XOS (ND‐XOS). Supplementation with RH‐XOS for 12 weeks successfully decreased the fasting plasma glucose, insulin, leptin, and LPS levels in DM‐XOS compared with DMC. Likewise, the insulin‐stimulated glucose uptake assessed by in vitro study was significantly enhanced in DM‐XOS, DMM, and DMM‐XOS. The diminished protein expressions of GLUT4 and pAkt^Ser473^ as well as pAMPK^Thr172^ were significantly modulated in DM‐XOS, DMM, and DMM‐XOS groups. Interestingly, RH‐XOS supplementation reversed the changed gut permeability, elevated the number of beneficial bacteria, both *Lactobacillus* and *Bifidobacterium* spp., and increased SCFAs production. Taken together, the results confirm the efficacy of RH‐XOS in achieving good glycemic control in diabetes by maintenance of gut microbiota and attenuation of endotoxemia. The findings reveal the benefits of RH‐XOS and open an opportunity to improve its value by its development as a nutraceutical for diabetes.

## Graphical abstract

The present study confirms the efficacy of RH‐XOS to achieve good glycemic control in diabetes by maintenance of endotoxemia and gut microbiota. The reduction of systemic endotoxemia may be contributed in an increased expression of the insulin‐stimulate glucose uptake protein markers, Akt^Ser473^ phosphorylation, and GLUT4 protein expression together with enhancement of the pAMPK activation resulting in stimulated glucose uptake and reduced hyperglycemia subsequently.

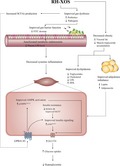

## 1 INTRODUCTION

Gut microbiota is the total number of microorganisms found in the human gastrointestinal tract, which make up 70% of the total microbes in the human body. The microbial cells in human gastrointestinal tract are estimated to be >100 trillion commensal organisms made up of over 1,000 different species (Brown, DeCoffe, Molcan, & Gibson, 2012). *Archaea*, fungi, yeast, and especially bacteria are the most dominant organisms in the human gut. *Firmicutes* and *Bacteroidetes* are the two most abundant bacterial phyla found in human intestinal microbiota, followed by *Proteobacteria*, *Actinobacteria*, *Fusobacteria*, and *Verrucomicrobia* phyla (Eckburg et al., 2005). Some microbes from these phyla are classified as saccharolytic bacteria (carbohydrate fermenting bacteria), which can produce short‐chain fatty acids (SCFAs) from nondigestible polysaccharides. SCFAs play a critical role in glucose, lipid, and cholesterol metabolism in various tissues (den Besten et al., 2013). The intestine is primarily colonized after birth by exposure to microorganisms through vaginal delivery, and these microorganisms participate in several physiological activities, such as supporting mucosal immune system, preserving metabolic homeostasis, and offering protection from pathogenic invasion (Thursby & Juge, 2017). Although there are personal and age‐dependent differences regarding the diversity of intestinal microbiota, a balance in microbial population in both number and type is generally obtained in the normal situation. The imbalance in intestinal microbiota is called gut dysbiosis, which directly influences the health status of the host (Claesson et al., 2012). There are several factors associated with altered intestinal microbiota, including radiation, stress, pathogenic infections, drugs, diets, and some toxins (Carding, Verbeke, Vipond, Corfe, & Owen, 2015; Cryan & O'Mahony, 2011; Hawrelak & Myers, 2004). Recent studies have illustrated that gut dysbiosis is associated with various diseases, such as CNS‐related disorders, inflammatory bowel disease, irritable bowel syndrome, celiac disease, colorectal cancer, metabolic disorders, obesity, and type 2 diabetes mellitus (T2DM) (Brown et al., 2012; Carding et al., 2015).

Insulin resistance is one of the typical risk factors that can lead to T2DM. In the last decade, several studies reported the relationship between insulin resistance, diabetes, and chronic low‐grade systemic inflammation (Castro, Macedo‐de la Concha, & Pantoja‐Meléndez, 2017; Liang, Hussey, Sanchez‐Avila, Tantiwong, & Musi, 2013; Nøhr et al., 2016). Although the occurrence of insulin resistance progression is not fully understood, low‐grade inflammation usually associated with people who are insulin resistance suggests that it plays a central role in T2DM (Nøhr et al., 2016). Unlike normal inflammation, low‐grade inflammation does not exhibit any general inflammatory symptoms, but it can be achieved by the typical inflammatory molecules and related signaling pathways that lead to the disease progression (Castro et al., 2017). Metabolic endotoxemia is characterized by elevated plasma lipopolysaccharide (LPS) level due to the outer membrane of gram‐negative bacteria, which is obtained from bacterial translocation that occur during increased intestinal permeability. LPS has been proposed as a possible mechanism of low‐grade systemic inflammation in T2DM (Liang et al., 2013).

Xylooligosaccharides (XOS) are considered as a class of prebiotics that stimulates the growth of beneficial bowel microbes or their activities to maintain the healthy state of the host. Several studies investigated the biological potentials of XOS in the past decade, for example, immunomodulatory, antibacterial, and antioxidant activities (Aachary & Prapulla, 2011; Kallel, Driss, Chaabouni, & Ghorbel, 2015). XOS also exhibited growth‐stimulating effect with respect to *Lactobacillus* and *Bifidobacterium* spp. both in in vitro and animal models (Christensen, Licht, Leser, & Bahl, 2014; Li, Summanen, Komoriya, & Finegold, 2015). In clinical trial, XOS consumption elevated the number of *Bifidobacterium* spp. in a dose‐dependent manner after 10 weeks of intervention, while the *Lactobacillus* counts, stool pH, SCFAs, and lactic acid had no statistically significant difference between placebo and intervention group (Finegold et al., 2014). Moreover, XOS also exhibited plasma glucose‐modulating effect and decreased other diabetes‐related risk factors, including HbA1c, cholesterol, low‐density lipoprotein cholesterol, oxidized low‐density lipoprotein, apolipoprotein B, fructosamine concentrations, and catalase activity, in type 2 diabetes subjects after supplementation with XOS for 8 weeks (Sheu, Lee, Chen, & Chan, 2008). However, the possible mechanisms of XOS on blood glucose modulation remain unclear.

Therefore, the present study aims to evaluate the antihyperglycemic effect of rice husk derived xylooligosaccharides (RH‐XOS) that was fed with a high‐fat diet (HFD) and streptozotocin (STZ)‐induced type 2 diabetic rats. Furthermore, this study elucidates the putative mechanisms regarding the effects of RH‐XOS on intestinal permeability, gut microbiota, and the protein expression of insulin signaling pathway in the skeletal muscle of type 2 diabetic rats.

## 2 MATERIALS AND METHODS

### 2.1 Rice husk derived xylooligosaccharides preparation, purification, and qualification

XOS production from rice husk was carried out according to a method previously described with some modifications (Khat‐Udomkiri et al., 2018). Rice husk was soaked in 12% NaOH solution at 120°C for 45 min with a liquid to solid ratio of 1:10. The supernatant was collected and acidified to a pH of 5 by adding glacial acetic acid. The alkali‐pretreated xylan was precipitated by adding three volumes of ice‐cold ethanol. The pellet was collected and dried in hot air oven until the dry pellet was obtained. The alkali‐pretreated xylan was dissolved in 50 mM of citric acid–Na_2_HPO_4_ buffer, pH: 6.0 (1% w/v). Then, the alkali‐pretreated xylan solution was incubated with commercial xylanase (Pentopan™ Mono BG) in a shaking incubator at 120 rpm, 50°C for 2 hr. At the indicated interval, enzyme reaction was stopped by boiling the extract on boiling water bath for 5 min. The crude XOS slurry was then separated using filter paper (Whatman No. 4). Thereafter, the liquid hydrolysate was demineralized using weak basic anion exchange resin (DOWEX™ Monosphere 66, Dow Chemical Company) and strong acidic cation exchange resin (DOWEX™ Monosphere 88 (H), Dow Chemical Company) according to the manufacturer's instructions. The clear XOS solution was concentrated using rotary vacuum evaporator at 60°C to obtain a semi‐purified XOS extract. The standardization of XOS extract was performed by HPLC employing a refractive index detector (Model 2,414, Waters Corporation), and ion exclusion chromatography column was used (Shodex SUGAR SH1011, SHOWA DENKO K.K.). The minimum quantity of XOS, which reached 270 mg/ml, was used in this study.

### 2.2 Animals, induction of diabetes, and experimental design

All experimental protocols were approved by the research animal ethical committee, Faculty of Pharmacy, Chiang Mai University, Thailand (approval no. 04/2015), in compliance with the National Institutes of Health guideline for the care and treatment of animals. Sixty male Wistar rats (150–200 g) were purchased from The National Laboratory Animal Center, Mahidol University, Thailand. All animals were housed under 12 hr of light/12 hr of darkness per day in a controlled temperature room (25 ± 2°C).

**Figure 1:**
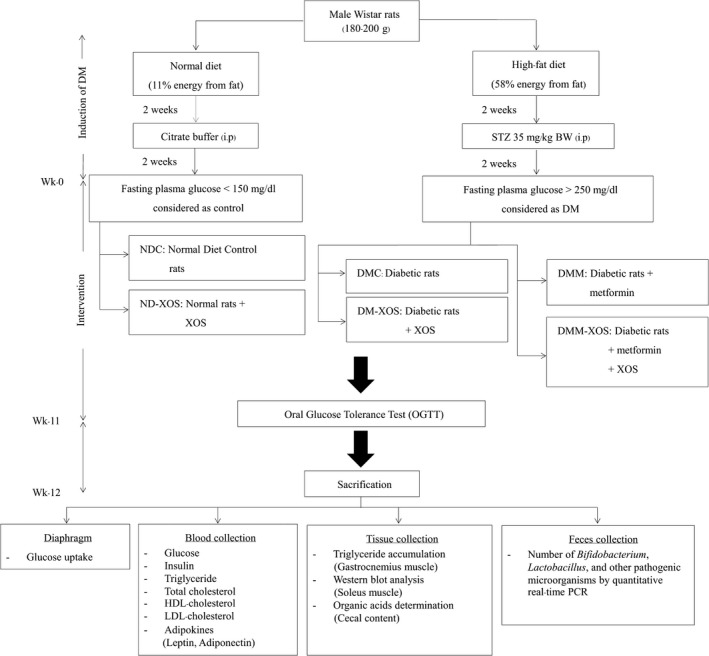
Schematic of overall experimental protocol . STZ: streptozotocin, BW: bodyweight, DM: diabetes mellitus, Wk; week, XOS; xylooligosaccharides

After the acclimatization period, animals were randomly divided into six groups as follows: normal diet control (NDC), normal rats supplemented with XOS at a dose of 500 mg/kg BW/day (ND‐XOS), T2DM rats (DMC), T2DM rats supplemented with XOS at a dose of 500 mg/kg BW/day (DM‐XOS), T2DM rats treated with metformin (DMM) at 30 mg/kg BW/day throughout the experimental period, and T2DM rats treated with a combination XOS and metformin at the same dose (DMM‐XOS). The DMM rats were considered as the positive control for standard medical treatment of this study. Normal diet rats were fed the standard pellet diet (11% energy from fat) (C.P. Mice Feed, S.W.T. Co., Ltd.) throughout the experiment. Type 2 diabetic rats were induced according to a method previously described (Srinivasan, Viswanad, Asrat, Kaul, & Ramarao, 2005). Briefly, the induction was carried out by the combination of HFD (58% energy from fat) for an initial period of 2 weeks and a single dose of STZ peritoneally injected (35 mg/kg BW). After 2 weeks of STZ injection, rats with a fasting plasma glucose level >250 mg/dl without hypoinsulinemia were classified as T2DM rat and used for further study. All rats had access to water and food ad libitum. Food intake and body weight were recorded weekly. Rats received XOS and metformin by oral gavage daily for 12 weeks. The oral glucose tolerance test (OGTT) was conducted a week prior to the end of the study. At week 12 of the study, the animals were sacrificed with an overdose of Nembutal® (Libourne). Blood and tissue samples were collected for further biochemical analyses. The gastrocnemius muscle and soleus muscle were collected and rapidly frozen in liquid nitrogen. Visceral fat was removed and weighted. Cecal content was collected and weighted after sacrificing by opening the abdominal wall, cutting off the cecum, manually squeezing the content into a sterile tube, and measuring the pH of the cecal content. Fecal samples were also collected at baseline (week 0) and after 12 weeks of intervention for fecal microbiota determination by quantitative real‐time PCR (q‐PCR). Blood, feces, cecal content, and all tissue samples were stored at –80°C for subsequent analysis. The overall animal protocol is presented in Figure 1.

### 2.3 Biochemical analyses

Plasma glucose, cholesterol, high‐density lipoprotein cholesterol (HDL‐C), low‐density lipoprotein cholesterol (LDL‐C), and triglyceride (TG) concentrations were assessed using commercial enzymatic colorimetric assay kits (Biotech, Thailand). Plasma insulin, leptin, and adiponectin concentrations were determined using a sandwich ELISA kit (LINCO Research).

The OGTT was carried out as previously described (Apichai, Pongchaidecha, Kaeapai, Jitprawet, & Lailerd, 2012). In brief, all animals were fasted overnight, and blood samples were collected as a baseline value (min‐0); after that, a glucose solution (2 g/kg BW) was administrated by oral gavage. Then, blood samples were collected at 15, 30, 60, and 120 min after glucose loading, and plasma glucose concentrations were measured using a commercial kit (Biotech, Thailand). The increments in plasma glucose concentration following glucose loading were expressed in terms of the area under the curve (AUC) for glucose, using the trapezoidal rule. The total AUC (TAUC) was calculated by the formula described below.

$$\frac{1}{2}\sum_{i=0}^{n-1}\left(t_{i+1}-t_{i}\right)\left(y_{i+1}+y_{i}\right)$$

The area below the baseline value was considered as the basal AUC (BAUC) value, which was calculated by multiplying the response at baseline of fasting glucose level ${(y}_{0})$ with 120 (total period of OGTT experiment). The area under the curve over the BAUC was defined as incremental AUC (IAUC) value and calculated by subtracting the area under the baseline from TAUC (TAUC ‐ BAUC or TAUC ‐($120\times y_{0}$)) (Matthews, Altman, Campbell, & Royston, 1990).

The degree of insulin resistance was determined by the homeostasis model assessment of insulin resistance (HOMA‐IR) index (Matthews et al., 1985). The HOMA‐IR index was calculated by the following formula:

$$\text{HOMA}\;-\;\text{IR index}\;=\;\frac{\text{fasting plasma insulin level}\;(\text{ng/ml})\;\times \;\text{fasting plasma glucose level}\;(\text{mg/dl})}{405.1}$$

### 2.4 Determination of muscle triglyceride content

The gastrocnemius muscle homogenate was prepared as described previously with slight modification (Frayn & Maycock, 1980). In brief, a 50–200 mg of muscle portion was minced and placed in a glass tube; 3 ml of chloroform–isopropanol mixture (2:3 v/v) was added into the glass tube, and the homogenate was transferred to a new glass tube and allowed to evaporate at 40°C for 16 hr to obtain the dried residue. The residue was dissolved and stirred in 10% bovine serum albumin (BSA). The triglyceride content was quantified using commercial colorimetric assay kits (Biotech, Thailand).

### 2.5 Measurement of in vitro glucose uptake in rat hemidiaphragm

The muscle glucose uptake was determined according to a previously described procedure with slight modification (Sabu & Subburaju, 2002). The rat diaphragm was removed with the avoidance of trauma, divided into two equal parts, and rinsed with cold balanced salt solution (BSS). The determination of glucose uptake was carried out by placing one part of the diaphragm in a conical flask containing 3 ml of BSS and glucose (2 mg/ml), while another part was placed in 3 ml of BSS with glucose (2 mg/ml) and insulin (0.25 IU/ml). Each hemidiaphragm was then incubated with carbogen (95% O_2_/5% CO_2_) with shaking (100 cycles/min) at 37°C for 90 min. After the incubation in glucose medium, the hemidiaphragms were removed, blotted with filter paper, and weighted. The remaining glucose concentration in the medium solution was quantified using commercial enzymatic colorimetric assay kits (Biotech, Thailand). The rate of glucose uptake was determined from the rate of decrease in glucose concentration in the media during incubation. The rate of glucose uptake was expressed as X mg of glucose per g of muscle tissue per 90 min incubation.

$$\text{Rate of glucose uptake}\;=\;\frac{\left(\text{Glucose level before incubation}\;-\;\text{Glucose level after incubation}\right)}{\text{Weight}\;\text{of diaphragm}}$$

### 2.6 Quantification of bacterial endotoxin in rat plasma

The plasma LPS, which indicated as gram‐negative bacterial endotoxin, was determined using QCL‐1000^TM^ Endpoint Chromogenic LAL Assay Kit (Lonza) as detailed in Jayashree et al. (2014). The absorbance of sample was quantified using spectrophotometer at 405–410 nm, and the result was expressed as EU/ml units.

### 2.7 Measurement of in vivo intestinal permeability

Fluorescein isothiocyanate‐dextran (FITC‐dextran) is one of the fluorescence molecules used for in vivo intestinal permeability according to previously published protocols (Cani et al., 2009; Joly Condette et al., 2014). After 12 weeks of intervention, rats were fasted overnight, and blood samples were collected as the negative control of the experiment to determine the background of rat plasma. Then, rats were administered 4,000 Da FITC‐dextran solution (600 mg/kg BW) (Sigma‐Aldrich) by oral gavage. Blood was collected 2.5 and 5 hr after FITC‐dextran administration. Plasma was separated immediately and diluted with an equal volume of PBS (pH 7.4). The release of FITC‐dextran in plasma sample was determined by a Synergy H4 hybrid reader (Bio‐Tek) at an excitation wavelength of 485 nm and emission wavelength of 535 nm and compared with standard curve of serially diluted FITC‐dextran.

### 2.8 Western blot analysis

The protein expression of insulin signaling cascade mediated glucose uptake was investigated as previously described (Apichai et al., 2012). The homogenization of soleus muscle was performed in six volumes of ice‐cold lysis buffer. After that, the homogenate was centrifuged at 5,000*× g* for 20 min at 4°C. The supernatant was used as the total cellular lysate. Then, the protein concentrations were determined with a Bradford protein assay reagent kit using BSA as a standard (Bio‐Rad). Aliquots of homogenates (35 µg) were subjected to protein separation by SDS‐PAGE on 10% sodium dodecyl sulfate–polyacrylamide gels and blotted onto nitrocellulose membrane using electroblotting system (Bio‐Rad). The nonspecific antibody binding to membrane was eliminated by blocking the membrane with Tris‐buffered saline with 5% low‐fat milk and 0.1% Tween 20 (TBST) at 4°C for 1 hr. The blotting membrane was then incubated with anti‐GLUT4 (Chemicon International), anti‐Akt (Millipore Corporation), antiphosphorylated‐Akt^Ser473^ (Millipore Corporation), anti‐AMPK (Millipore Corporation), and antiphosphorylated‐AMPK^Thr172^ (Millipore Corporation) at 4°C overnight. GADPH antibody was used as the loading control for this experiment. The membrane was washed and incubated with a goat monoclonal anti‐rat IgG antibody conjugated to horseradish peroxidase (Chemicon International). Immunoreactive bands were visualized on Kodak Hyperfilm (Kodak) using an enhanced ECL kit (GE Healthcare). The band density was quantified with a densitometer using Scion Image software. The concentration of protein was expressed as the relative protein compared with the value of the control group, which was arbitrarily set as 100. The level of the phosphorylated signaling element was expressed relative to the total amount of that protein from the same sample.

### 2.9 DNA extraction from fecal samples

Bacterial DNA was collected from fecal samples (60–70 mg) using NucleoSpin^®^ DNA stool kit (Macherey‐Nagel) following the manufacturer's instructions. Qualitative analysis of purified bacterial DNA was done by SPECTROstar Nano Absorbance microplate reader (BMG Labtech).

### 2.10 q‐PCR assay conditions and cycle threshold

q‐PCR analyses were carried out as previously described with some modifications (Keenan et al., 2013) in 96‐well optical plates on the Quantstudio TM6 Flex Real‐Time PCR System (Applied Biosciences). The amplification reaction was performed in a total volume of 20 µl containing 10 µl SYBR master mix, 2 µl fecal bacterial DNA sample, 1 µl reverse primer, 1 µl forward primer, and 6 µl deionized water. The group‐specific primers of bacterial targets based on 16S rDNA sequences are listed in Table S1. q‐PCR was conducted as follows: UDG activation step at 50°C for 2 min followed by initial denaturation at 95ºC for 2 min, 40 cycles of denaturation step at 95°C for 20 s, and the annealing/extension step at 60°C for 20 s. Melting curve analysis was performed after each run to check the nonspecific amplification of the primers. The cycle threshold (Ct) of bacterial DNA was calculated by absolute quantification strategy using the standard curve of the target bacterial strain. The result was expressed as log CFU/ml.

### 2.11 Measurement of organic acids in cecal content

The organic acids (acetic, propionic, butyric, and lactic acids) in the cecal content were assessed by high‐performance liquid chromatography (HPLC) as described previously (Pattananandecha et al., 2016). Briefly, sample homogenate was prepared in 0.15 mM sulfuric acid and centrifuged at 10,000×* g* at 4°C for 10 min. The supernatant was collected and filtered through 0.22 µm nylon syringe filter. The samples were analyzed by Shimadzu‐HPLC system using Shodex SUGAR SH1011 (SHOWA DENKO K.K.). The detection system was carried out using UV detector at 210 nm, and column temperature was maintained at 75°C. Samples were isocratically eluted with 5 mM sulfuric acid at 0.6 ml/min. The concentration of organic acids was quantified by comparison with standard curve, and the results were expressed as µmol/g of sample.

### 2.12 Statistical analysis

The SPSS Advanced Statistics software (version 17 SPSS Inc.) was used for statistical analysis. Data were presented as mean value ± standard error of the mean (*SEM*). One‐way analysis of variance (ANOVA) followed by LSD’s post hoc analysis was used to determine significant differences between groups. For all statistical analysis, a *p*‐value of < .05 was considered to be a significant difference.

## 3 RESULTS

### 3.1 Effect of RH‐XOS on metabolic parameters of HFD‐STZ‐induced T2DM rats

The characteristics and metabolic parameters of the experimental rats are shown in Table 1. The initial body weight of the animals before RH‐XOS or metformin supplement was not statistically significantly different between the groups, except the initial body weight of DM‐XOS group. The combination of high‐fat diet and low‐dose STZ injection significantly increased the body weight, weight gain, as well as visceral fat/body weight (VF/ 100 g BW) compared with normal control rats (*p* < .05). These data show that visceral obesity was found in the diabetic rats.

**Table 1:** General characteristics and metabolic parameters of RH‐XOS, metformin, and RH‐XOS combined with metformin supplement in normal and T2DM rats (means ± *SEM* from 8–10 animals per group)

	NDC	ND‐XOS	DMC	DM‐XOS	DMM	DMM‐XOS
General Characteristics						
Initial BW (g)	394.50 ± 7.47	376.00 ± 7.22	389.50 ± 5.98	364.44 ± 9.59*, **	383.75 ± 7.78	373.50 ± 10.22
Final BW (g)	577.00 ± 16.75	529.50 ± 14.54	718.50 ± 27.65*	608.89 ± 25.24**	630.71 ± 27.87**	608.50 ± 33.03**
Weight gain (g)	182.50 ± 16.06	153.50 ± 17.13	342.22 ± 24.92*	244.44 ± 26.20*, **	247.86 ± 24.92*, **	235.00 ± 27.47*, **
VF/100 g BW	8.13 ± 0.22	6.90 ± 0.56	12.93 ± 0.49*	8.56 ± 0.48**	10.54 ± 0.41*, **	9.80 ± 0.48*, **
Metabolic parameters						
Initial plasma glucose (mg/dl)	139.33 ± 7.64	138.91 ± 5.17	315.03 ± 26.15*	303.64 ± 18.28*	314.44 ± 23.12*	316.67 ± 25.13*
Final plasma glucose (mg/dl)	142.69 ± 11.36	120.03 ± 4.31	371.15 ± 18.97*	168.92 ± 7.63**	195.11 ± 12.19*, **	191.69 ± 10.03*, **
Plasma insulin (ng/ml)	5.09 ± 0.75	5.43 ± 0.75	9.91 ± 1.30*	5.75 ± 0.50**	7.43 ± 0.60*, **	5.54 ± 0.85**
HOMA‐IR	1.89 ± 0.34	1.53 ± 0.19	9.17 ± 1.32*	2.40 ± 0.25**	3.63 ± 0.44*, **	2.57 ± 0.46**
Plasma leptin (ng/ml)	14.04 ± 1.93	14.73 ± 2.66	32.72 ± 3.00*	17.86 ± 1.52**	19.90 ± 4.12**	17.98 ± 1.83**
Plasma adiponectin (ng/ml)	203.65 ± 11.65	208.28 ± 13.19	182.93 ± 1.50	223.62 ± 12.44**	208.29 ± 13.21	216.44 ± 11.87
Plasma TG (mg/dl)	28.60 ± 2.61	33.75 ± 3.87	96.42 ± 8.28*	40.20 ± 3.95**	29.90 ± 2.08**	36.13 ± 4.56**
Plasma total cholesterol (mg/dl)	44.38 ± 2.99	39.61 ± 2.22	80.49 ± 6.99*	48.46 ± 5.07**	33.99 ± 3.17**	29.65 ± 2.56*, **
Plasma HDL‐C (mg/dl)	55.50 ± 2.60	89.50 ± 10.10*, **	52.50 ± 0.29	72.00 ± 2.89*, **	76.00 ± 2.30*, **	66.00 ± 3.46
Plasma LDL‐C (mg/dl)	12.00 ± 1.15	18.50 ± 0.87*	29.50 ± 1.44*	15.00 ± 0.58*, **	16.50 ± 0.87*, **	14.00 ± 1.73**

Abbreviations: BW, body weight; HDL‐C, high‐density lipoprotein cholesterol; HOMA‐IR, homeostasis model assessment of insulin resistance; LDL‐C, low‐density lipoprotein cholesterol; LPS, lipopolysaccharide; TG, triglyceride; VF, visceral fat.

* *p* < .05 indicates the significant differences from NDC.

** *p* < .05 indicates the significant differences from DMC.

At the end of the study, the fasting plasma glucose concentration of DMC group was significantly increased (17.7%) compared with the initial value (Table 1). Moreover, significantly increased plasma insulin, leptin, and HOMA‐IR levels in addition to decreased plasma adiponectin level were noticed in the DMC group compared with the NDC group. The plasma lipid profiles (TG, cholesterol, LDL‐C) were significantly higher in the DMC group than in the NDC group (*p* < .05). These results confirm that the T2DM rats used in this study showed general characteristics of T2DM, hyperglycemia, hyperlipidemia, and insulin resistance, which are close to the characteristics of T2DM patients.

Supplementation with RH‐XOS, metformin, and a combination of RH‐XOS and metformin significantly reduced body weight, weight gain, and VF/100 g BW compared with the DMC group (Table 1, *p* < .05). To explore the antidiabetic effect of RH‐XOS, the plasma biochemical parameters were measured. As shown in Table 1, the RH‐XOS supplement did not alter the fasting plasma glucose levels among normal rats. Type 2 diabetic rats showed a significantly higher fasting plasma glucose level compared with normal rats (*p* < .05). Supplementation with RH‐XOS at a dose of 500 mg/kg BW successfully reduced the fasting plasma glucose levels in type 2 diabetic rats (–44%, *p* < .05). Diabetic rats treated with metformin and a combination of RH‐XOS and metformin also showed significantly reduced fasting plasma glucose level (–38% and –40% respectively, *p* < .05). To determine whether the glucose‐lowering effect of RH‐XOS is involved in pancreatic insulin secretion or insulin sensitivity, the fasting plasma insulin levels and HOMA‐IR were measured. At the end of the experiment, the fasting plasma insulin levels in the DM‐XOS, DMM, and DMM‐XOS groups were significantly decreased compared with the DMC group (*p* < .05). Therefore, the decreased fasting plasma glucose levels in type 2 diabetic rats treated with RH‐XOS might be due to the improvement in insulin sensitivity, similar to metformin treatment. In accordance with these results, the DM‐XOS and DMM‐XOS groups recorded significantly reduced HOMA‐IR compared with the DMC group (*p* < .05). In addition, HOMA‐IR was significantly decreased in the DMM group compared with the DMC group (*p* < .05). These findings demonstrate that supplementation with RH‐XOS reduced the whole‐body insulin resistance in T2DM rats, which is similar to the case of type 2 diabetic rats treated with metformin.

To evaluate the relationship between the adipocyte‐derived hormone and insulin sensitivity in this experimental model, plasma adiponectin and leptin levels were determined. As shown in Table 1, the plasma leptin level was significantly increased in the DMC group compared with the NDC group (*p* < .05), while the plasma adiponectin level was lower than that of the NDC group. Interestingly, supplementation with RH‐XOS, metformin, and a combination of RH‐XOS and metformin significantly decreased the plasma leptin levels compared with the DMC group (*p* < .05). However, only RH‐XOS supplement significantly increased the plasma adiponectin level in type 2 diabetic rats (*p* < .05).

In the present study, we also examined the hypolipidemic effect of RH‐XOS on type 2 diabetic rats, and the results are shown in Table 1. In normal rats, supplementation with RH‐XOS did not alter the plasma triglyceride and total cholesterol levels. The plasma lipids, triglyceride, total cholesterol, and LDL‐C levels were significantly higher in the DMC group than in the NDC group (*p* < .05). Remarkably, supplementation with RH‐XOS, metformin, and a combination of RH‐XOS and metformin significantly reduced the plasma triglyceride, total cholesterol, and LDL‐C levels in type 2 diabetic rats compared with the DMC group (*p* < .05). In addition, significant augmentation of the plasma HDL‐C levels was observed in the DM‐XOS and DMM groups (*p* < .05).

**Figure 2:**
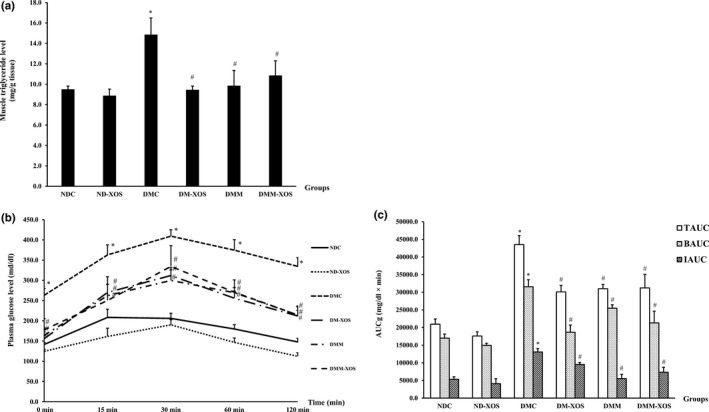
Triglyceride level in gastrocnemius muscle (a), plasma glucose responses (b), and area under the curve for glucose (AUC_g_) (c) from the oral glucose tolerance test (OGTT) after RH‐XOS, metformin, and RH‐XOS combined with metformin supplement for 12 weeks. TAUC: total area under the curve; BAUC: basal area under the curve; IAUC: incremental area under the curve. Data are represented as means ± *SEM* from five animals per group; *p < .05 indicates the significant differences from NDC, and ^#^ *p* < .05 indicates the significant differences from DMC

As shown in Figure 2a, there was no difference in triglyceride accumulation in the gastrocnemius muscle between the NDC and ND‐XOS groups. On the other hand, the accumulation of skeletal muscle triglycerides was significantly higher in the DMC group than in the NDC group (*p* < .05). A significant reduction of skeletal muscle triglyceride contents was found in the DM‐XOS, DMM, and DMM‐XOS groups compared with the DMC group (*p* < .05).

To determine whether RH‐XOS can affect the whole‐body insulin sensitivity in type 2 diabetic rats, the OGTT was performed. As shown in Figure 2b, there were no significant differences between the plasma glucose levels at all time points in the NDC and ND‐XOS groups. In addition, the TAUC, BAUC, and IAUC in the NDC and ND‐XOS groups were comparable (Figure 2c). The plasma glucose levels before and after glucose loading revealed significantly higher values in the DMC group at all time points in comparison to the NDC group (Figure 2b). Compared with the NDC group, the total area under the curve (TAUC), basal area under the curve (BAUC), and incremental area under the curve (IAUC) were markedly increased in the DMC group (*p* < .05). Notably, the plasma glucose levels were significantly lower in the DM‐XOS, DMM, and DMM‐XOS groups at 30, 60, and 120 min after glucose loading compared with the DMC group (*p* < .05) as shown in Figure 2b. There was significant reduction in the TAUC, BAUC, and IAUC values in the DM‐XOS, DMM, and DMM‐XOS groups compared with the DMC group (Figure 2c, *p* < .05). These results suggest that supplementation with RH‐XOS at a dose of 500 mg/kg BW effectively improved glucose tolerance in T2DM rats, which is similar to the findings of the treatment with metformin.

### 3.2 Effect of RH‐XOS on glucose uptake by isolated rat hemidiaphragm

**Figure 3:**
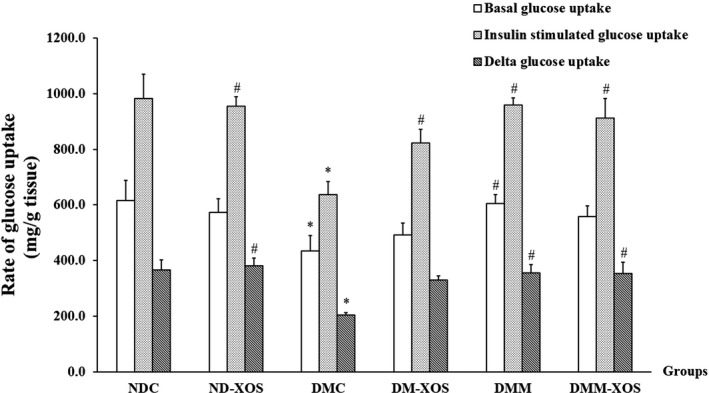
Glucose uptake by isolated rat hemidiaphragm after RH‐XOS, metformin, and RH‐XOS combined with metformin supplement for 12 weeks. Data are represented as means ± *SEM* from five animals per group; *p < .05 indicates the significant differences from NDC, and ^#^ *p* < .05 indicates the significant differences from DMC

To examine whether the RH‐XOS had any effect on the skeletal muscle glucose transport system, the basal and insulin‐stimulated glucose uptake in the isolated diaphragm were determined. Isolated rat hemidiaphragm was used as the candidate skeletal muscle for glucose uptake under in vitro conditions. As illustrated in Figure 3, significantly decreased basal glucose uptake, insulin‐stimulated glucose uptake, and delta glucose uptake were found in the DMC group compared with the NDC group (*p* < .05). After 12 weeks of intervention, metformin treatment exhibited significantly increased basal glucose uptake, insulin‐stimulated glucose uptake, and delta glucose uptake (*p* < .05) compared with the DMC group. Interestingly, the insulin‐stimulated glucose uptake of RH‐XOS and RH‐XOS combined with metformin supplementation in T2DM rats were significantly improved compared with the DMC group (Figure 3, *p* < .05). In addition, the delta glucose uptake was significantly elevated in the hemidiaphragm of the ND‐XOS and DMM‐XOS groups (*p* < .05). These findings indicate that RH‐XOS supplementation enhanced the skeletal glucose uptake due to insulin stimulation in the isolated hemidiaphragm from type 2 diabetic rat. Also, these findings were accompanied by an improvement in insulin sensitivity.

### 3.3 Effect of RH‐XOS on plasma endotoxin in T2DM rats

**Figure 4:**
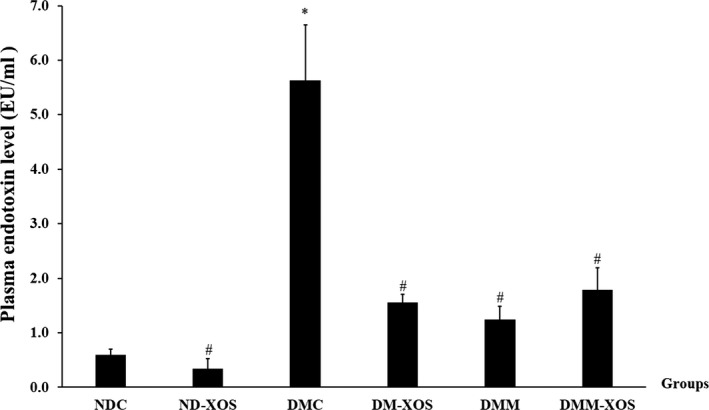
Plasma endotoxin level after RH‐XOS, metformin, and RH‐XOS combined with metformin supplement for 12 weeks. Data are represented as means ± *SEM* from five animals per group; *p < .05 indicates the significant differences from NDC, and ^#^ *p* < .05 indicates the significant differences from DMC

The presence of circulating LPS was identified as metabolic endotoxemia, which is related to several diseases, especially obesity and T2DM. The plasma LPS levels of all experimental groups are shown in Figure 4. At the end of the study, the plasma LPS level in diabetic control rats significantly increased compared with normal control rats (*p* < .05). The administration of RH‐XOS, metformin, and RH‐XOS combined with metformin for 12 weeks significantly lessened the increased plasma LPS levels in diabetic rats (*p* < .05). These results provide evidence that RH‐XOS consumption could help to attenuate endotoxemia in T2DM rats.

### 3.4 Effect of RH‐XOS on intestinal permeability in T2DM rats

**Figure 5:**
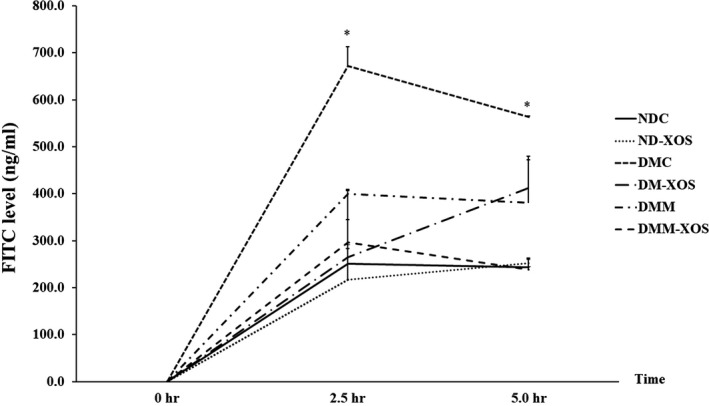
Time course of 4 kDa FITC‐dextran permeability of normal and diabetic rats after RH‐XOS, metformin, and RH‐XOS combined with metformin supplement for 12 weeks Data are represented as means ± *SEM* from four animals per group; *p < .05 indicates the significant differences from NDC, and ^#^ *p* < .05 indicates the significant differences from DMC; FITC, fluorescein isothiocyanate

Paracellular transport is one of the key characteristics of several diseases, including diabetes mellitus. Various substances are used for measurement of changes in paracellular permeability. In this study, fluorescein isothiocyanate‐dextran (FITC‐Dextran) was selected for determination of intestinal paracellular permeability. The consumption of HFD and low‐dose STZ injection successfully elevated the plasma FITC‐Dextran concentration after 2 and 5 hr of loading period (*p* < .05) compared with rats fed on the normal diet (Figure 5). This result confirms that disruption of intestinal permeability occurred in the T2DM rat model used in this study. After 12 weeks of intervention, there were no statistically significant differences in the plasma FITC‐Dextran concentration at all time points for DM‐XOS, DMM, and DMM‐XOS groups compared with the NDC group. The findings suggest that impaired intestinal paracellular permeability was attenuated by RH‐XOS consumption.

### 3.5 Effect of RH‐XOS on insulin signaling and glucose transport in skeletal muscle of T2DM rats

**Figure 6:**
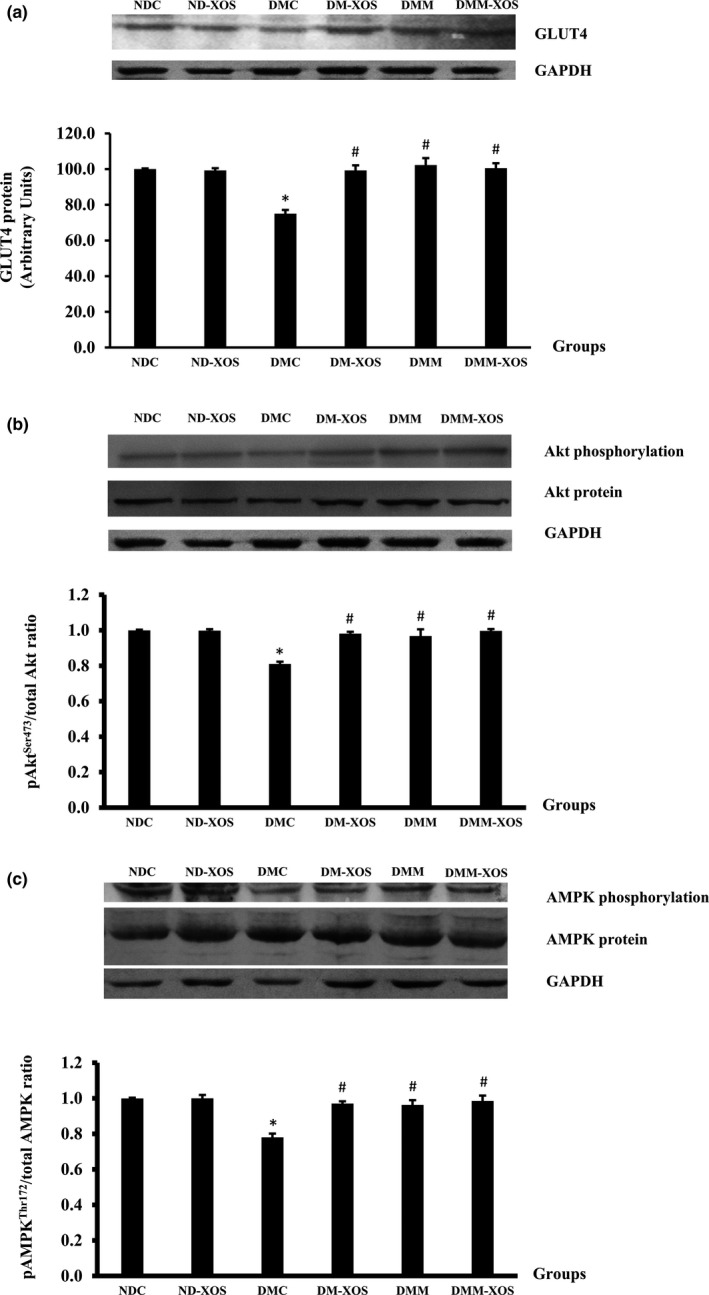
Expression of GLUT4 (a), Akt and Akt^Ser473^ phosphorylation (b), and AMPK and AMPK^Thr172^ phosphorylation (c) determined by Western blot in soleus muscle after RH‐XOS, metformin, and RH‐XOS combined with metformin supplement for 12 weeks. Data are represented as means ± *SEM* from 5–8 animals per group; *p < .05 indicates the significant differences from NDC, and ^#^ *p* < .05 indicates the significant differences from DMC; GLUT4, glucose transporter 4

To verify the underlying mechanisms behind the possible beneficial effects of RH‐XOS regarding the improvement of insulin‐stimulated glucose uptake, we examined whether RH‐XOS can improve insulin signaling cascade in the skeletal muscle of type 2 diabetic rats. As the activation of Akt plays an important role in insulin signaling for GLUT4 translocation, we examined the phosphorylation of this downstream insulin signaling protein. There was no significant difference in GLUT4 expression in soleus muscle between the NDC and ND‐XOS groups (Figure 6a). As expected, the expression of GLUT4 protein in the DMC group was significantly lower than that of the NDC group (*p* < .05). The administration of RH‐XOS, metformin, and RH‐XOS combined with metformin for 12 weeks successfully increased GLUT4 protein expression in the soleus muscle (*p* < .05) compared with the NDC group (Figure 6a). The expressions of Akt protein in all experimental groups were comparable. In normal rats, supplementation with RH‐XOS did not alter the ratio of pAkt^Ser473^/Akt protein in comparison to the NDC group (Figure 6b). As illustrated in Figure 6b, the ratio of pAkt^Ser473^/Akt protein was significantly lowered in the DMC compared with the NDC groups (*p* < .05). Interestingly, supplementation with RH‐XOS, metformin as well as a combination of metformin and RH‐XOS significantly increased pAkt^Ser473^/Akt protein ratio compared with the DMC group (*p* < .05).

In addition to the insulin signaling proteins, the phosphorylation of AMPK is known to stimulate GLUT4 translocation to the plasma membrane via the insulin‐independent pathway in the skeletal muscle. Therefore, we further investigated whether RH‐XOS can induce the phosphorylation of AMPK^Thr172^. As shown in Figure 6c, the pAMPK^Thr172^/AMPK protein ratio in the NDC group was comparable to the value in the ND‐XOS group. A significant reduction in pAMPK^Thr172^/AMPK protein ratio was observed in the DMC group in comparison to the NDC group (*p* < .05). Compared with the DMC group, the pAMPK^Thr172^/AMPK protein ratio in all groups of RH‐XOS supplementation in diabetes and the DMM group was significantly elevated (*p* < .05). However, the protein expressions of AMPK were not significantly different in all experimental groups (Figure 6c).

### 3.6 Effect of RH‐XOS on cecal weight, cecal pH, and organic acids

**Figure 7:**
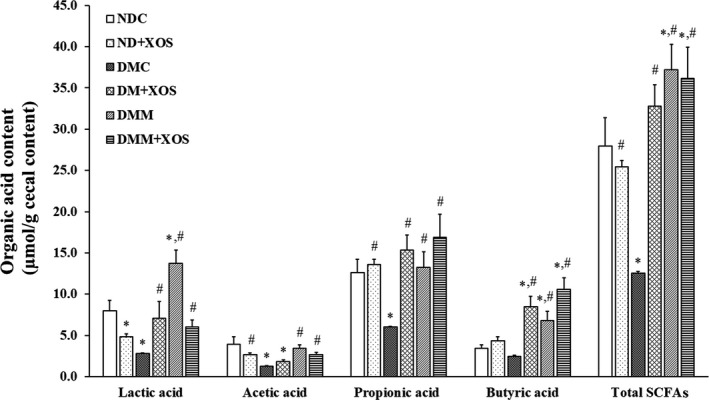
The level of organic acids in the cecal content after RH‐XOS, metformin, and RH‐XOS combined with metformin supplement for 12 weeks. Data are represented as means ± *SEM* from five animals per group; *p < .05 indicates the significant differences from NDC, and ^#^ *p* < .05 indicates the significant differences from DMC

As shown in Table 2, diabetic rats in DMC, DM‐XOS, DMM, and DMM‐XOS groups exhibited significantly lower cecal weight compared with normal control rats (*p* < .05), while normal rats fed with RH‐XOS showed increased cecal weight compared with the DMC group (*p* < .05). No significant difference in cecal pH was observed in all treatment groups. However, decreased cecal pH was found in the ND‐XOS (−3.33% vs. NDC), DM‐XOS (−6.67% vs. NDC, −3.45% vs. DMC), and DMM‐XOS (−7.14% vs. NDC, −10.34% vs. DMC) groups. Next, the organic acids in the cecal content were investigated to confirm the occurrence of end products after carbohydrate fermentation in rat colon, as shown in Figure 7. The results showed that the butyrate concentration in diabetic rats fed with RH‐XOS, metformin, and RH‐XOS combined with metformin was significantly higher compared with normal and diabetic control rats (*p* < .05). Significantly decreased propionate, acetate, lactate, and total SCFAs contents were found in the DMC compared with the NDC group (*p* < .05). The administration of RH‐XOS, metformin, and RH‐XOS combined with metformin showed significantly increased propionate concentration in the ND‐XOS, DM‐XOS, DMM, and DMM‐XOS groups compared with the DMC group (*p* < .05). The normal rats fed with RH‐XOS showed significantly lower lactate levels in the cecal content compared with normal rats (*p* < .05). The acetate concentrations in the ND‐XOS, DMM, and DMM‐XOS groups were significantly higher compared with diabetic control rat (*p* < .05). The administration of metformin to diabetic rats significantly increased the lactic acid content compared with the NDC and DMC groups (*p* < .05); furthermore, a significantly increased lactate level was found in RH‐XOS combined with metformin fed diabetic rats compared with the DMC group (*p* < .05). However, DMM‐XOS significantly decreased lactate level compared with DMM group (−56.13% vs. DMM, *p* < .05). The concentration of total SCFAs was significantly increased in the ND‐XOS and DM‐XOS groups compared with the diabetic control rats (*p* < .05); especially, the DMM and DMM‐XOS group exhibited a significantly increased level compared with both the NDC and DMC groups (*p* < .05). These findings indicate that RH‐XOS can decrease the cecal pH of diabetic and control rats. Additionally, the administration of RH‐XOS increased SCFAs content, especially propionate and butyrate, in the cecal content of normal, diabetic, and metformin‐treated diabetic rats.

**Table 2:** Cecal weight and cecal pH of RH‐XOS, metformin, and RH‐XOS combined with metformin supplement in normal and T2DM rats (means ± *SEM* from 5 animals per group)

	NDC	ND‐XOS	DMC	DM‐XOS	DMM	DMM‐XOS
Cecal weight (g)	3.69 ± 0.53	4.49 ± 0.54**	1.44 ± 0.15*	2.30 ± 0.65*	1.68 ± 0.14*	1.94 ± 0.39*
Cecal pH	7.50 ± 0.29	7.25 ± 0.48	7.25 ± 0.25	7.00 ± 0.71	7.75 ± 0.25	6.50 ± 0.50

* *p* < .05 indicates the significant differences from NDC.

** *p* < .05 indicates the significant differences from DMC.

### 3.7 Effect of RH‐XOS on fecal microbiota

**Figure 8:**
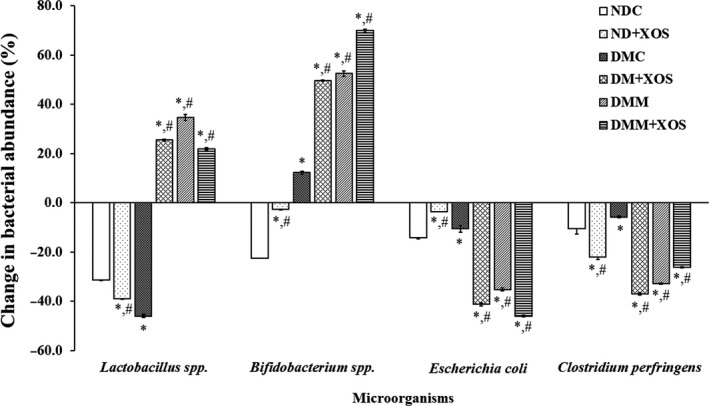
Mean percent change in bacterial abundance from baseline analyzed by q‐PCR in feces after RH‐XOS, metformin, and RH‐XOS combined with metformin supplement for 12 weeks. Data are represented as means ± *SEM* from five animals per group; *p < .05 indicates the significant differences from NDC, and ^#^ *p* < .05 indicates the significant differences from DMC

The number of fecal *Lactobacillus* spp. was significantly decreased in the ND‐XOS and DMC groups (−38.98% and −46.04% from baseline, respectively) compared with the rats fed with the normal diet (*p* < .05), as shown in Figure 8. After the intervention period, the mean change in fecal *Lactobacillus* spp. in diabetic rats fed with RH‐XOS, metformin, and a combination of RH‐XOS and metformin was significantly higher compared with normal and diabetic control rats (*p* < .05). However, the amount of *Bifidobacterium* spp. in the feces of diabetic control rats was significantly increased (12.32% from baseline) compared with the respective control rats (*p* < .05). The reduced number of *Bifidobacterium* spp. was successfully addressed after RH‐XOS administration in normal rats (−2.54% from baseline, *p* < .05). Also, a significant increase in *Bifidobacterium* abundance was observed in diabetic rats fed with RH‐XOS, metformin, and a combination of RH‐XOS and metformin compared with the respective control rats (*p* < .05). The mean percentage change of *E. coli* and *C. perfringens* in normal rats fed with RH‐XOS was significantly (*p* < .05) decreased (−3.53% and −22.06% from baseline) compared with normal control rats, respectively. There was a statistically significant reduction (*p* < .05) in the mean percentage change of *E. coli* and *C. perfringens* in diabetic control rats in comparison to normal control rats. The administration of RH‐XOS, metformin, and the combination of RH‐XOS and metformin caused significant reduction in comparison to normal and diabetic control groups (*p* < .05). These results suggest that RH‐XOS administration increases the abundance of *Lactobacillus* and *Bifidobacterium* spp. from baseline in diabetic and metformin‐treated diabetic rats. Meanwhile, the suppression of *E. coli* and *C. perfringens* growth was facilitated by RH‐XOS consumption for 12 weeks in normal, diabetic, and metformin‐treated diabetic rats.

## 4 DISCUSSION

The major challenge in this study is to determine whether XOS extracted from rice husk (RH) can ameliorate hyperglycemia in STZ‐induced type 2 diabetic rats fed with a high‐fat diet. The outcomes of the study provide evidence that demonstrates the antihyperglycemic effect of RH‐XOS partly due to the attenuation of insulin resistance and an improvement in muscle glucose uptake as well as insulin signaling in type 2 diabetic rat model. Its antihyperglycemic effect may lie in its ability to modulate endotoxemia and gut microbiota.

Diabetes is defined as abnormalities in blood sugar control overtime, which is characterized by hyperglycemia as a consequence of total lack of insulin secretion or lack of insulin activity in target tissues or both (American Diabetes Association, 2014). The development of new naturally derived products for patients with diabetes has become an interesting field. In this study, daily consumption of RH‐XOS at a dose 500 mg/kg BW for 12 weeks exhibited the beneficial effects of lowering plasma glucose level and normalizing triglyceride, cholesterol, and LDL‐C levels in T2DM rats, which indicated its antihyperglycemia and hypolipidemia effects.

Nowadays, several animal models have been established for diabetes research. Streptozotocin (STZ), a natural glucosamine‐nitrosourea substance, has been used for induction of diabetes in both rats and mice for a decade. After STZ injection, the destruction of pancreatic β‐cell is achieved through alkylation in the diabetogenic activity of STZ, which results in decreased insulin production and leads to uncontrollable plasma glucose level in rats (Junod et al., 1967; Szkudelski, 2001). High‐fat diet along with low‐dose STZ treatments are generally used for T2DM rat induction (Srinivasan et al., 2005). In the present study, the general characteristics exhibited by the T2DM rats are similar to those in humans, including abdominal obesity, insulin resistance, hyperglycemia, hyperinsulinemia, and dyslipidemia. Therefore, these rats could be used to establish a rat model of T2DM and were suitable for the purpose of studying antidiabetic effects in our experimental study.

Rice husk is one of the agricultural wastes obtained from rice milling process in Thailand (Khat‐Udomkiri et al., 2018). The disposal of agricultural wastes by burning causes air pollution and respiratory morbidity and mortality (Arbex et al., 2012; Tipayarom & Oanh, 2007). Based on our previous study, RH‐XOS extraction is a sustainable strategy to resolve this issue. Presently, there are several reports indicating the biological activities of XOS (Aachary & Prapulla, 2011; Kumar, Pushpa, & Prabha, 2012). Previously, the administration of commercially available XOS in STZ‐induced diabetic rats improved several morbidities, including suppression of the elevated serum glucose level, serum cholesterol and triglyceride, and triglyceride composition after 5 weeks of intervention. Additionally, XOS significantly improved both cecal content and cecal acetate level in STZ‐induced diabetic rats (Gobinath, Madhu, Prashant, Srinivasan, & Prapulla, 2010; Imaizumi, Nakatsu, Sato, Sedarnawati, & Sugano, 1991). Similarly, our present study exhibits the beneficial effects of RH‐XOS on fasting plasma glucose level, triglyceride, cholesterol, and HOMA‐IR in HFD‐STZ‐induced T2DM rats. In a clinical study, the administration of XOS (4 g/day) for 8 weeks showed beneficial effects on blood sugar, HbA1c, fructosamine, and lipid profile in T2DM (Sheu et al., 2008). These data support the assertion that RH‐XOS has antihyperglycemia and antihyperlipidemia effects and improves insulin resistance.

Although several reports mentioned that XOS has beneficial effects on diabetes, both in vivo and clinical studies, the underlying mechanisms remain unclear. To explore the possible antidiabetic mechanisms of XOS, the skeletal glucose uptake was investigated using isolated rat hemidiaphragm. Skeletal muscle is known as the major disposal tissue of glucose (Lailerd, Saengsirisuwan, Sloniger, Toskulkao, & Henriksen, 2004). We speculate that the attenuation of plasma glucose level in HFD‐STZ‐induced T2DM rats could be a consequence of glucose transportation activity in the skeletal muscle. According to our results, RH‐XOS and RH‐XOS combined with metformin supplementation significantly improved insulin‐stimulated glucose uptake in T2DM rats. However, the direct effect of RH‐XOS and other prebiotics on glucose transport in T2DM rats was not investigated. A study demonstrated that increased glucose uptake was observed after treatment with metabolites produced by probiotic *Lactobacillus* increased glucose accumulation in Caco‐2 cells (Rooj, Kimura, & Buddington, 2010). Consistently, cell‐free extract of *Bifidobacterium lactis* HY8101 treatment increased glucose accumulation into TNF‐α‐induced insulin‐resistant rat skeletal muscle cells (Kim et al., 2014). Glucose transporter 4 (GLUT4) is a major glucose transporter protein that facilitates glucose uptake into skeletal muscle, improves insulin sensitivity, and contributes to control of whole‐body glucose homeostasis (Huang & Czech, 2007). Similar to our finding, previous studies have shown that a decrease in GLUT4 protein expression was exhibited in STZ‐induced diabetic rats (Apichai et al., 2012; Keapai, Apichai, Amornlerdpison, & Lailerd, 2016; Sunil, Duraipandiyan, Agastian, & Ignacimuthu, 2012). Interestingly, RH‐XOS‐treated T2DM rats had significantly modulated total GLUT4 protein expression compared with T2DM control group. Translocation of cytosolic GLUT4 to plasma membrane was mediated by Akt activation (Taniguchi, Emanuelli, & Kahn, 2006). Remarkably, RH‐XOS supplementation for 12 weeks in T2DM rats significantly restored insulin‐stimulated Akt^Ser473^ phosphorylation in skeletal muscle, which is consistent with the results from a previous in vitro study that reported the activation of Akt phosphorylation of insulin‐resistant C2C12 cells after incubation with inulin (Yun et al., 2009). Moreover, glucose uptake, GLUT4 protein expression in skeletal muscle, and Akt phosphorylation in liver were increased in STZ‐induced diabetic mice after treatment with either lotus seed oligomeric procyanidins or a combination of XOS and *Bifidobacterium animalis* for 12 weeks (Li, Sui, Wu, Xie, & Sun, 2017). Several studies reported that glucose transport into skeletal muscle is also facilitated by an insulin‐independent mechanism via AMPK activation (Cao et al., 2012; Takikawa, Inoue, Horio, & Tsuda, 2010). As expected, the present study demonstrated that the phosphorylation of AMPK^Thr172^ was markedly reduced in T2DM rats, and these alterations were restored after RH‐XOS, metformin, and the combination of RH‐XOS and metformin treatments. To date, metformin is known as an antidiabetic medication for T2DM patients, and its mechanism to control blood sugar is mainly activated by GLUT4 translocation via AMPK pathway (Lee et al., 2012). Although there are a few reports on the relationship between XOS and their AMPK activation, supplementation with inulin, a well‐known prebiotic, demonstrated the activation of AMPK phosphorylation in C2C12 myotubes (Yun et al., 2009). These data suggest that either insulin‐stimulated Akt phosphorylation or AMPK activation by RH‐XOS treatment leads to increase in GLUT4 translocation and enhanced glucose transport into skeletal muscle, which lessens hyperglycemia in T2DM rats.

Several reports mentioned the relationship between excessive ingestion of high‐fat diet and gut microbiota dysbiosis, which results in insulin resistance and finally develops to T2DM. Low‐grade inflammation was initiated by the alteration of intestinal microbiota, especially gram‐negative bacteria‐derived LPS, which increased metabolic endotoxemia through the impaired intestinal barrier function and leads to chronic systemic inflammation development via LPS/TLR4 signal transduction pathway (Han & Lin, 2014; Thiennimitr et al., 2018). Significant elevation in LPS, ZO‐1, TNF‐α, and IL‐6 levels was reported in T2DM patients compared with healthy subjects (Jayashree et al., 2014). Likewise, STZ‐induced diabetic rats showed significantly increased serum lipoperoxide levels and LPS‐induced serum TNF‐α activities after 24 weeks of experiment (Qiang et al., 1998). These results are quite similar to our findings shown in Figure 4. Significantly reduced endotoxin levels in RH‐XOS, metformin, and RH‐XOS combined with metformin supplementations in T2DM rats were demonstrated compared with DMC. Our finding is similar to an earlier report that treatment with XOS in obese insulin‐resistant rats reduced LPS level in the serum and proinflammatory cytokines gene expression, resulting in beneficial effect on insulin resistance (Thiennimitr et al., 2018). In contrast to our finding, a previous study showed that XOS did not improve intestinal barrier function in normal rats (Christensen et al., 2014); however, our results found that impaired intestinal barrier function was improved after RH‐XOS, metformin, and RH‐XOS combined with metformin administration in T2DM rats. It was proposed that the maintenance of gut homeostasis by restoring beneficial intestinal microbes, especially *Lactobacillus* and *Bifidobacterium* spp., may be an effective treatment for T2DM. Subsequently, we determined the effect of RH‐XOS, metformin, and RH‐XOS combined with metformin supplementation on gut dysbiosis. The results indicate that administration of RH‐XOS, metformin, and RH‐XOS combined with metformin stimulated the growth of beneficial bacteria, including *Lactobacillus* and *Bifidobacterium* spp. in T2DM rats. Importantly, RH‐XOS also markedly inhibited the growth of representative pathogenic gut microorganisms, *E. coli* and *C. perfringens*, compared with the DMC group (Figure 8). In agreement with our findings, XOS was reported to have prebiotic properties, both in in vivo and clinical studies (Hsu, Liao, Chung, Hsieh, & Chan, 2004; Lin et al., 2016; Thiennimitr et al., 2018). SCFAs are substances obtained after fermentation of dietary fibers by intestinal microbiota, and they play an important role in regulation of glucose metabolism. Increased amount of SCFAs, especially acetate and butyrate, might improve glucose uptake by increasing the expression of GLUT4 and fatty acid oxidation in skeletal muscle through pAMPK activation (Canfora, Jocken, & Blaak, 2015). In this study, RH‐XOS supplementation increased the SCFAs in the cecal content along with the increased expression of AMPK phosphorylation and GLUT4 protein expression, leading to enhanced skeletal glucose uptake and attenuated muscle triglyceride. The modulation of endotoxemia and amelioration of gut dysbiosis might be the main mechanism that fully explains the antihyperglycemic effect of RH‐XOS.

**Figure 9:**
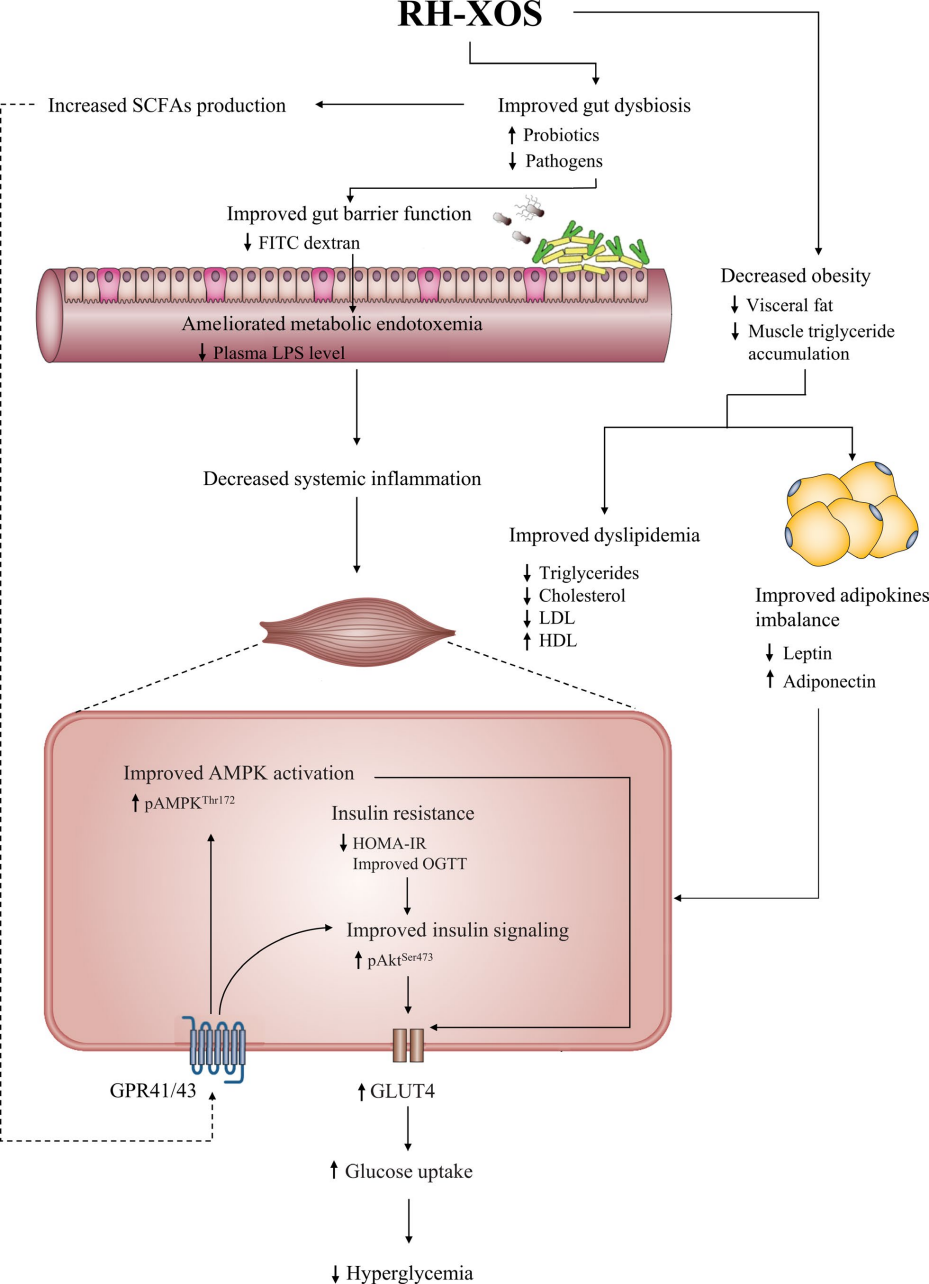
The proposed mechanism of antihyperglycemic action of RH‐XOS in T2DM rats which modified from Canfora et al. (2015)

In conclusion, this study, firstly, revealed that oral administration of RH‐XOS has a significant antidiabetic potential in HFD‐STZ‐induced type 2 diabetic rat model. RH‐XOS has the potential to regulate blood glucose level and improve glucose tolerance, insulin resistance, leptin resistance, as well as dyslipidemia. Furthermore, we demonstrated the underlying mechanisms of RH‐XOS in modulating gut microbiota, which lead to increased amount of SCFAs in the cecal content and reduces systemic endotoxemia. The reduction in systemic endotoxemia may contribute to an increased expression of insulin‐stimulated glucose uptake protein markers, Akt^Ser473^ phosphorylation, and GLUT4 protein expression, in addition to the enhancement of the pAMPK activation, resulting in stimulated glucose uptake and reduced hyperglycemia subsequently (Figure 9). These findings indicate an opportunity to improve the value of RH‐XOS by its development as a nutraceutical for diabetes.

## CONFLICT OF INTEREST

The authors confirm that there are no known conflicts of interest.

## ETHICAL APPROVAL

All animals were housed and cared for in accordance with the rules and regulations of the Animal Research Committee of Faculty of Pharmacy, Chiang Mai University, Thailand (approval no. 04/2015), in compliance with the National Institutes of Health guideline for the care and treatment of animals.
