# Supplementary material for: Antihyperglycemic effect of rice husk derived xylooligosaccharides in high‐fat diet and low‐dose streptozotocin‐induced type 2 diabetic rat model
Source: Food Sci Nutr. 2019 Dec 9;8(1):428–44. doi: 10.1002/fsn3.1327 (PMC6977422; doi:10.1002/fsn3.1327)
Supplement: Supplementary file 1 [file FSN3-8-428-s001.docx]

**Supporting Information**

**Table S1** Group-specific primers of bacterial targets based on 16 S rDNA sequences

| Organisms | Forward Primer | Reverse Primer | Reference |
| --- | --- | --- | --- |
| *Lactobacillus* group | 5’-AGCAGTAGGGAATCTTCCA-3’ | 5’-ATTYCACCGCTACACATG-3’ | Ignacio et al., 2016 |
| *Bifidobacterium* group | 5’-TCGCGTCCGGTGTGAAAG-3’ | 5’-CCACATCCAGCATCCAC-3’ | Rinttilä, Kassinen, Malinen, Krogius, & Palva, 2004 |
| *Enterobacteriaceae* | 5’-CATTGACGTTACCCGCAGAAGAAGC-3’ | 5’-CTCTACGAGACTCAAGCTTGC-3’ | Bartosch, Fite, Macfarlane, & McMurdo, 2004 |
| *Clostridium* sp. group | 5’-GCACAAGCAGTGGAGT-3’ | 5’-CTTCCTCCGTTTTGTCAA-3’ | Larsen et al., 2010 |

**REFERENCES**

Bartosch, S., Fite, A., Macfarlane, G. T ., & McMurdo, M. E. T . (2004 ). Characterization of bacterial communities in feces from healthy elderly volunteers and hospitalized elderly patients by using real‐time PCR and effects of antibiotic treatment on the fecal microbiota. Applied and Environmental Microbiology , 70 (6), 3575–3581. https://doi.org/10.1128/aem.70.6.3575-3581.2004

Ignacio, A., Fernandes, M. R., Rodrigues, V. A., Groppo, F. C., Cardoso, A. L., Avila‐Campos, M. J., & Nakano, V. (2016). Correlation between body mass index and faecal microbiota from children. Clinical Microbiology and Infection, 22 (3), 258.e251–258. https://doi.org/10.1016/j.cmi.2015.10.031

Larsen, N., Vogensen, F. K., van den Berg, F. W. J., Nielsen, D. S., Andreasen, A. S., Pedersen, B. K., … Jakobsen, M. (2010). Gut microbiota in human adults with type 2 diabetes differs from non‐diabetic adults. PLoS One , 5(2), e9085. https://doi.org/10.1371/journal.pone.0009085

Rinttilä, T ., Kassinen, A., Malinen, E., Krogius, L., & Palva, A. (2004). Development of an extensive set of 16S rDNA‐targeted primers for quantification of pathogenic and indigenous bacteria in faecal samples by real‐time PCR. Journal of Applied Microbiology , 97 (6), 1166–1177. https://doi.org/10.1111/j.1365-2672.2004 .024 09.x
